# Stage-specific differential gene expression profiling and functional network analysis during morphogenesis of diphyodont dentition in miniature pigs, Sus Scrofa

**DOI:** 10.1186/1471-2164-15-103

**Published:** 2014-02-06

**Authors:** Fu Wang, Jing Xiao, Wei Cong, Ang Li, Fulan Wei, Junji Xu, Chunmei Zhang, Zhipeng Fan, Junqi He, Songlin Wang

**Affiliations:** 1 Molecular Laboratory for Gene Therapy & Tooth Regeneration, Beijing Key Laboratory of Tooth Regeneration and Function Reconstruction, School of Stomatology, Capital Medical University, Tian Tan Xi Li No.4, Beijing 100050, PR China; 2 Department of Oral Basic Science, College of Stomatology, Dalian Medical University, Liaoning 116044, China; 3 Laboratory of Molecular Signaling and Stem Cells Therapy, Beijing Key Laboratory of Tooth Regeneration and Function Reconstruction, School of Stomatology, Capital Medical University, Beijing 100050, China; 4 Department of Biochemistry and Molecular Biology, School of Basic Medical Sciences, Capital Medical University, Beijing 100069, China

**Keywords:** Gene expression profile, Diphyodont, Odontogenesis, Miniature pig

## Abstract

**Background:**

Our current knowledge of tooth development derives mainly from studies in mice, which have only one set of non-replaced teeth, compared with the diphyodont dentition in humans. The miniature pig is also diphyodont, making it a valuable alternative model for understanding human tooth development and replacement. However, little is known about gene expression and function during swine odontogenesis. The goal of this study is to undertake the survey of differential gene expression profiling and functional network analysis during morphogenesis of diphyodont dentition in miniature pigs. The identification of genes related to diphyodont development should lead to a better understanding of morphogenetic patterns and the mechanisms of diphyodont replacement in large animal models and humans.

**Results:**

The temporal gene expression profiles during early diphyodont development in miniature pigs were detected with the Affymetrix Porcine GeneChip. The gene expression data were further evaluated by ANOVA as well as pathway and STC analyses. A total of 2,053 genes were detected with differential expression. Several signal pathways and 151 genes were then identified through the construction of pathway and signal networks.

**Conclusions:**

The gene expression profiles indicated that spatio-temporal down-regulation patterns of gene expression were predominant; while, both dynamic activation and inhibition of pathways occurred during the morphogenesis of diphyodont dentition. Our study offers a mechanistic framework for understanding dynamic gene regulation of early diphyodont development and provides a molecular basis for studying teeth development, replacement, and regeneration in miniature pigs.

## Background

Odontogenesis is driven by many genes encoding signature and signaling molecules, which are regulated by epithelial-mesenchymal interactions mediated by the fine-tuning of conserved signaling pathways including Shh, Wnt, FGF, Tgf-β, Bmp, Eda, etc. [1,2]. The current understanding of the molecular mechanisms controlling tooth development has come mostly from studies in mice, which have only one set of non-replaced dentition with a diastema and are obviously different from humans with respect to tooth anatomy and development; therefore, relatively little is known about the mechanisms of tooth replacement in mammals [2-5]. A desirable model remains a significant obstacle for understanding the mechanisms of tooth replacement. Pigs resemble humans in anatomy, physiology, pathophysiology, development, and immune responses [6-8], and have been successfully used as an experimental model for craniofacial research [9-18]. Recently, swine have become more popular as a useful pre-clinical model for jaw osteoradionecrosis, jaw bone defects, salivary gland radiation damage, periodontal diseases, craniofacial disorders, temporal mandibular joint fractures, and autoimmune diseases [9-13]. Swine would serve as excellent pre-clinical experiment alternatives for tooth development and regeneration compared with the rodent models widely used. The initiation, eruption time, and sequence of tooth development in miniature pigs are quite similar in humans. In addition, swine have diphyodont dentition, which is an excellent model for studying teeth replacement [18-22]. The teeth anatomy and deciduous teeth development of miniature pigs have been described previously [20,21,23]. To date, there is a lack of gene expression and regulation profiles during odontogenesis in swine and the differences in gene expression profiles between swine, rodents, and human remain largely undefined. Recently, the porcine genome project was completed [24-33]; thus, further analysis of the mechanisms of morphogenetic patterns and diphyodont replacement should be possible using molecular methods in this large animal model.

**Figure 1:**
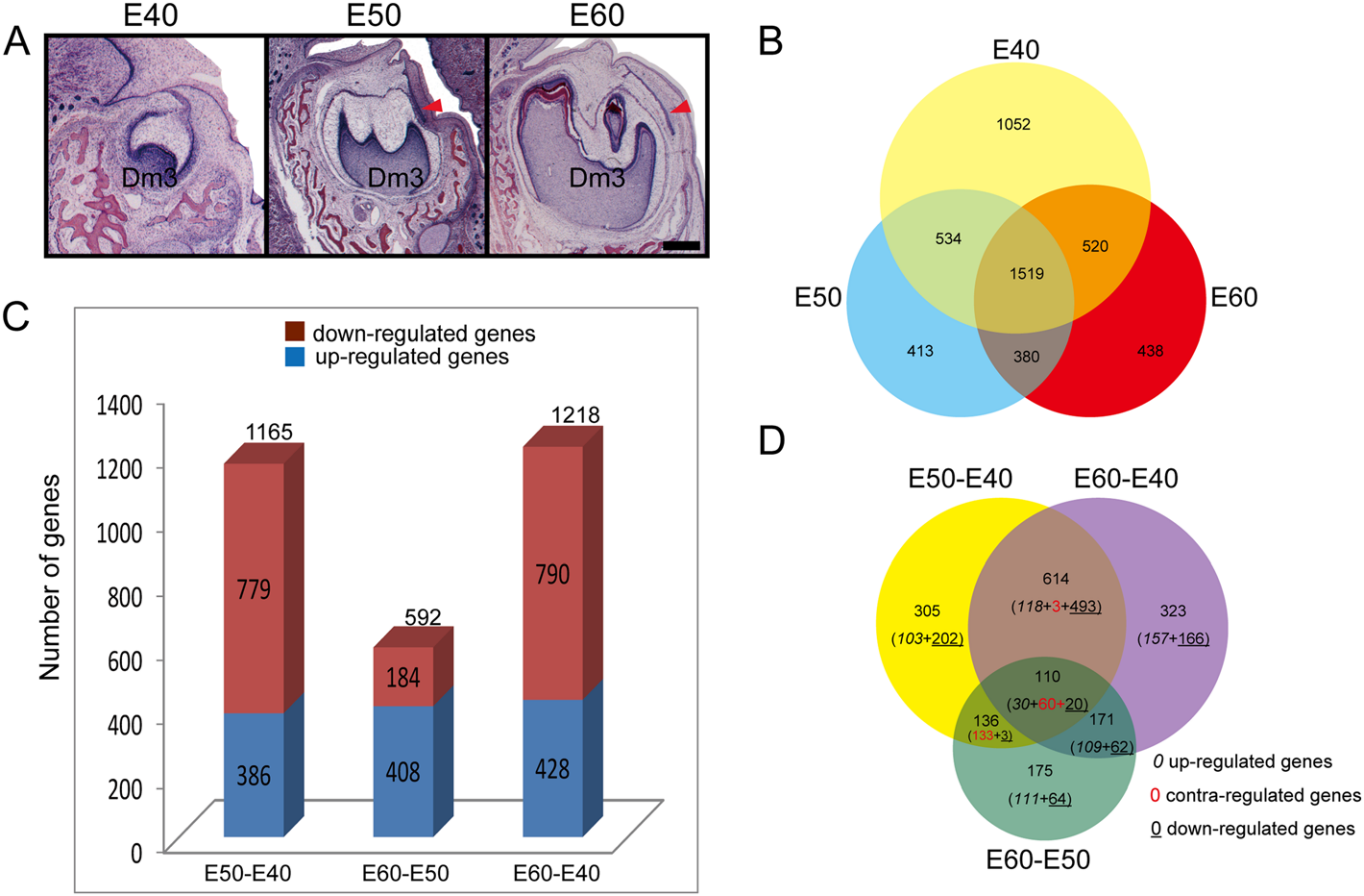
**Gene expression profiles during early morphogenesis of deciduous molars and successional dental lamina. (A)** The third deciduous molar (Dm3) in the mandible of the miniature pig is in the cap stage at E40 (no successional dental lamina), bell stage at E50 (lingual successional dental lamina appearing, red arrowhead), and secretory stage at E60 (successional dental lamina changes little). Scale bar, 500 μm. **(B)** Venn diagrams showing the stage-specific gene expression profiling at E40, E50, and E60, with each section showing the gene expression number. **(C)** The genes with differential expression between stages, with down-regulated genes being predominant on E50 versus E40, while up-regulated genes were more dominant when comparing E60 versus E50. **(D)** Venn diagrams showing the numbers and type (up-, down- or contra-regulated) of transcriptional alterations between stages.

The primary teeth and successional counterparts of Wuzhishan miniature pigs have many similarities to humans in size, morphology, number, and form of teeth [20,21]. We previously determined the patterns of early diphyodont morphogenesis in the Wuzhishan miniature pig [34]. The results indicated that the last deciduous molar (third deciduous molar, Dm3) in the mandible of the miniature pig undergoes cap stage at embryonic day 40 (E40), bell stage at E50, secretory stage at E60, and its successional tooth is derived from the lingual successional dental lamina (sdl), which is visible at E50 when its deciduous counterpart is in bell stage, and changes little in morphology at E60 (Figure 1A). The developmental features offer a desirable pattern for understanding diphyodont replacement. Moreover, the analysis of the genome sequence of the Wuzhishan miniature pig has been published [33]. Based on the above studies, we used the Wuzhishan miniature pig as a large animal model to investigate the gene expression profiles and functional regulation networks of developing deciduous molars and their replacement counterparts using microarrays. We analyzed the differential gene expression profiles and characterized the gene regulatory networks of morphogenesis and cell differentiation during early diphyodont morphogenesis. We identified the stage-specific genes and pathways with differential regulation. The results provide a molecular base for further research on tooth development, replacement, and regeneration using the miniature pig as a large animal model.

## Results and discussion

### Global transcriptome complexity and gene expression profiles during early morphogenesis of deciduous molars and successional dental lamina

After digital processing of the array data, 18,528 transcripts (79.67% of all probe sets) were present in the tooth germs from the three stages, of which 17,297, 16,518, and 16,529 probes were expressed on E40, E50, and E60, respectively. Of these, 1,052, 413, and 438 genes were expressed only in tooth germs on E40, E50, and E60, respectively, and 1519 genes were expressed in all three stages (Figure 1B). Large numbers of transcripts were differentially expressed with more than two-fold changes between any two given stages, and 2,035 genes with annotations were screened for subsequent analysis (Additional file 1). The number of genes with significant differential expression varied between the three stages investigated: 1,165 between E50 and E40 (386 up-regulated genes, 779 down-regulated genes), 592 between E60 and E50 (408 up-regulated genes, 184 down-regulated genes), and 1,352 between E60 and E40 (428 up-regulated genes, 790 down-regulated genes) (Figure 1C). Venn diagram analysis showed the most changes in gene expression occurred on E60 versus E40 (323 genes were significantly altered, with 157 up-regulated and 166 down regulated). Less transcripts were significantly altered between E60 and E50 (175 genes were significantly altered, with 111 up-regulated and 64 down regulated) [35]. In addition, there were a total of 110 genes with significant alterations during all 3 stages, with 30 genes commonly up-regulated, 20 commonly down-regulated, and 60 contra-regulated (diverse transcription) (Figure 1D). The results suggested the differential gene expression patterns in the different developmental stages of the pig tooth germ were consistent with the transitions of morphogenesis. During early diphyodont morphogenesis from E40 to E60, the Dm3 underwent 2 stages (“cap stage to bell stage” and “bell stage to secretory stage”). The Dm3 showed dramatic changes in morphology at E50, which reached bell stage with characteristic sdl for secondary permanent tooth, and Dm3 morphogenesis changed little at E60 (secretory stage) without evidence of morphological changes in the sdl. So, the more changes in transcription between E50 and E40 implied that more gene variations were involved in Dm3 morphogenesis from cap stage to bell stage as well as the appearance of sdl The results also showed the genes were predominantly down-regulated on E50 versus E40; while, up-regulated genes dominated the morphogenesis of tooth germs on E60 versus E50.

In summary, the gene expression profiles indicated that spatio-temporal patterns of gene expression were mainly down-regulated; while, both activation and inhibition occurred during diphyodont morphogenesis. Our results provide a molecular basis for studying teeth development, replacement, and regeneration and a better understanding of the dynamic gene regulation of early diphyodont development in the miniature pig.

### Immunocytochemistry and real-time RT-PCR verification of gene expression

**Figure 2:**
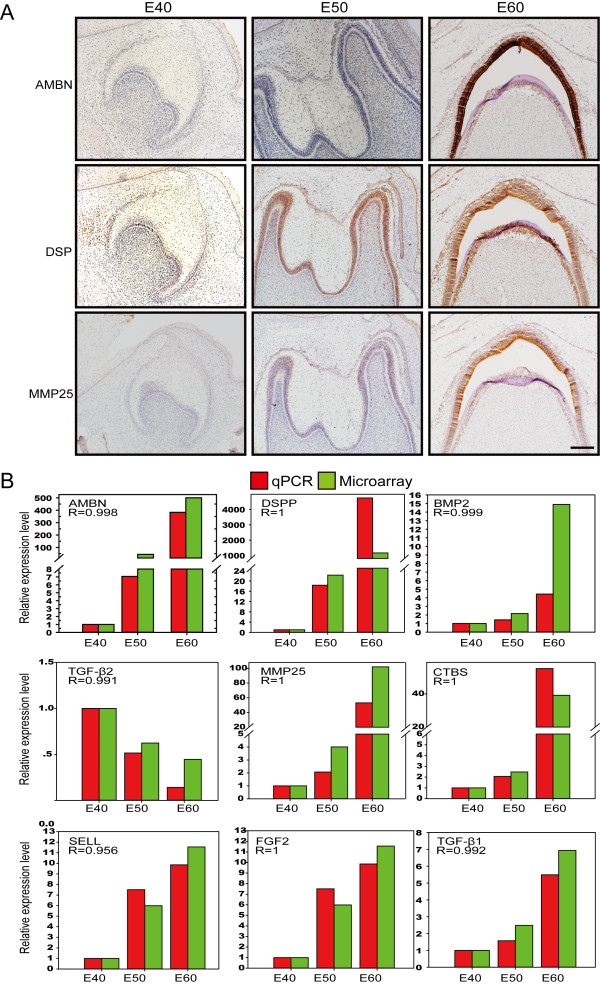
**Verification of gene expression by immunocytochemistry and real-time RT-PC. (A)** Immunocytochemistry assay visualized by DAB (brown) showing the expression changes of Ambn (ameloblastin), Dsp (dentin sialoprotein), and MMP25 (matrix metallopeptidase 25) were in concordance with the results of the normalized microarray. **(B)** Pearson correlation analysis (R > 0.9, *p <* 0.05) showing the expression levels of nine genes detected by real-time RT-PCR (qPCR, red box) were in agreement with the results of the normalized microarray (green box). R, Pearson correlation coefficient.

Validation of microarray data was achieved using immunocytochemistry and real-time reverse transcription–polymerase chain reaction (qRT-PCR). We used immunocytochemistry to detect proteins known to be important for tooth germ cell differentiation (i.e., Ambn, Dsp, and MMP25). The results confirmed there were similar changes in expression at the protein level (Figure 2A). Nine representative genes were chosen for qRT-PCR, based either on their fold change and/or their potential functions. The qRT-PCR results were also consistent with the normalized microarray data (Figure 2B).

### Gene ontology analysis of screened genes

Gene ontology (GO) analysis of significantly up-regulated differential genes on E50 versus E40 showed (Additional file 2) these genes strongly correlated with multicellular organism development, odontogenesis of dentine-containing teeth, regulation of gene expression, cell adhesion, signal transduction, integrin-mediated signaling pathways, metabolic processes, and apoptosis. The down-regulated differential genes were mainly involved in muscle organ development (Additional file 2). Significantly up-regulated differential genes on E60 versus E50 correlated strongly with biomineral tissue development, cell redox homeostasis, metabolic processes, cell adhesion, ion transport, multicellular organism development, oxidation-reduction, epithelial cell maturation, apoptosis, integrin-mediated signaling pathways, and the odontogenesis of dentin-containing teeth (Additional file 3). The down-regulated differential genes were mainly involved in metabolic process, signal transduction, ion transport, muscle contraction, blood vessel development, epithelial to mesenchymal transition, inflammatory response, and immune response (Additional files 2 and 3).

**Figure 3:**
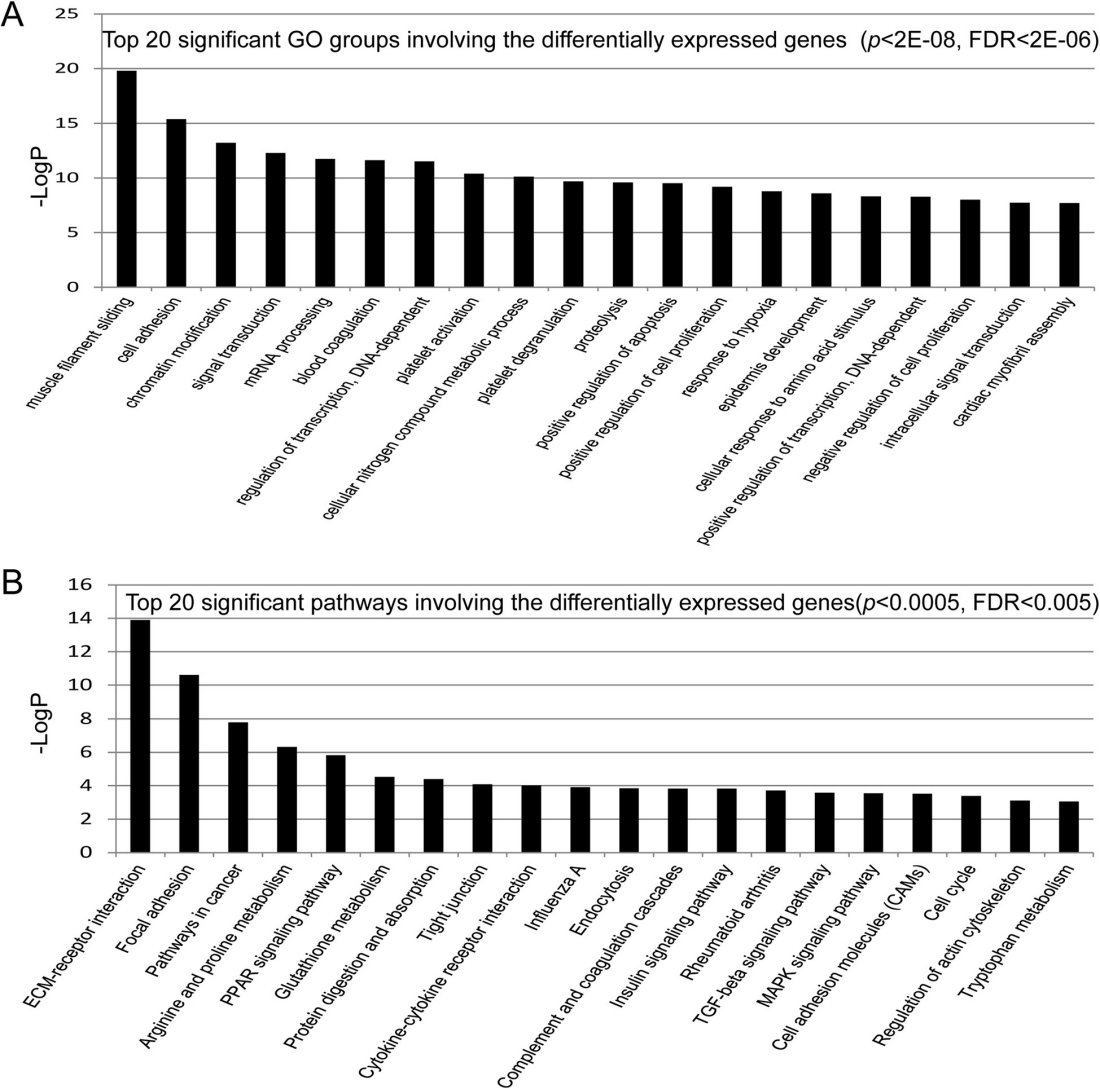
**Gene ontology and pathway analysis of differential genes. (A)** Top 20 significant GO groups involving the differentially expressed genes (*p <* 2E-08, FDR < 2E-06). **(B)** Top 20 significant pathways involving the differentially expressed genes (*p <* 0.0005, FDR < 0.005). The greater –Log*p*, the higher the significance.

The enriched GO analyses of all significant differentially expressed genes between any two stages were associated with 110 categories (*p <* 0.0002 and FDR < 0.01, Additional file 4). There were 20 very significant GO categories (Figure 3A, Additional file 4) filtered by *p*-value and FDR (*p <* 1E-8, FDR < 1E-8), with the most significant (*p <* 2E-12, FDR < 6E-10) being “muscle filament sliding”, “cell adhesion”, “chromatin modification”, “signal transduction”, and “mRNA processing”, which correlated with down-regulated genes (Figure 3A, Additional file 4). We also identified the specific genes that significantly correlated with odontogenesis among the enriched GO categories, including odontogenesis of dentine-containing teeth (*AMELX, PDGFRA, LRP6, BMP7, CA2, WNT6, MSX1, BMP2, AMBN*), tooth mineralization (*AMELX, COL1A1*), regulation of odontogenesis of dentine-containing teeth (*IFT88, DICER1*), and positive regulation of tooth mineralization (*WNT6, AMELX*) (Additional file 5). Furthermore, recent studies suggested dental lamina is a source of odontogenic stem cells, which play an important role in tooth replacement [2,22,36-41]. Our results showed the enriched genes linked to the GO term molecular function of epithelial development (Additional file 6), including Wnt and BMP, were confirmed to be involved in tooth replacement in other animals, such as snakes, alligators, and ferrets, which suggests signal molecule conservative in evolution and species [2,37,38,42,43]. These enriched genes and GO groups should be studied further to determine regulation patterns of diphyodont replacement in a large animal model.

### Pathway analysis of screened genes

Pathway analysis was used to determine significant pathways involving the differential genes, according to KEGG, Biocarta and Reatome. A total of 50 significant pathways involved differential genes (*p <* 0.05, Additional file 7). There were 20 very significant pathways (*p <* 0.0005, FDR < 0.005) (Figure 3B), with the most significant (*p <* 0.00001, FGR < 0.0001) being ECM-receptor interactions, focal adhesion, pathways in cancer, arginine and proline metabolism, and the PPAR signaling pathway (Figure 3B, Additional file 7), some of which were closely correlated with down-regulated genes consistent with down-regulated genes dominating the morphogenesis above mentioned. For example, the ECM-receptor interaction and focal adhesion pathways are involved in homeostasis. The extracellular matrix (ECM), including the integrin and collagen families, serves an important role in tissue and organ morphogenesis and in the maintenance of cell and tissue structure and function. The cells and ECM interactions play essential roles in important biological processes including cell adhesion, cell migration, cell survival and apoptosis, cell proliferation, cell differentiation, and regulation of gene expression. In addition, integrins function as mechanoreceptors and provide a force-transmitting physical link between the ECM and cytoskeleton [44-48]. Taking into account importance of dental lamina on successional odontogenesis, we matched the genes correlated with epithelium determination derived from enriched GO categories with 50 significant pathways above-mentioned, 4 possible pathways were enriched to contribute most likely to diphyodont dentition, including TGF-beta signaling pathway, MAPK signaling pathway, Cytokine-cytokine receptor interaction and Hedgehog signaling pathway (Additional files 6 and 7). This prediction is reflected in the subsequent path-net constructed.

### Series test of cluster (STC) and STC-GO analysis of genes

**Figure 4:**
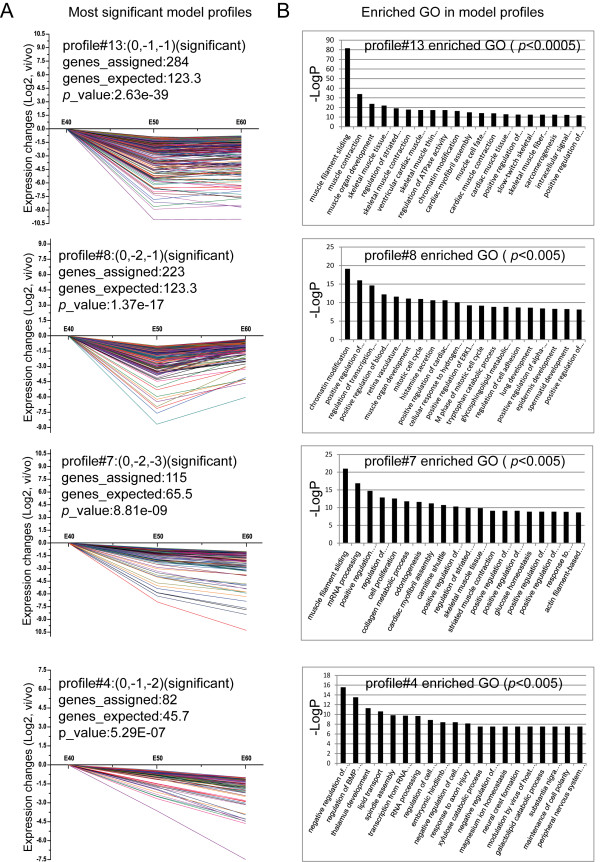
**STC-GO analysis. (A)** The most significant gene expression tendencies of profiles 4, 7, 8, and 13 showed predominantly down-regulation patterns of gene expression. The horizontal axis represents stages, and the vertical axis shows the time series of gene expression levels for the gene after Log normalized transformation. The value in brackets after the profile represents the variation intensity, the genes assigned represent the gene number in each profile, the genes expected represent the theoretical gene number in each profile, and the *p*-value indicates significance. **(B)** The significant enriched GO terms in profiles 4, 7, 8, and 13.

**Figure 5:**
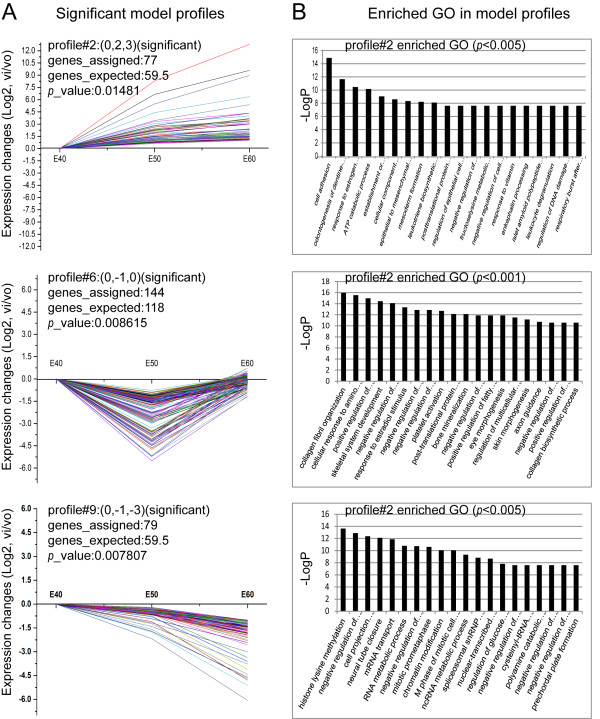
**Significant gene expression tendencies and GO enrichment of profiles 2, 6 and 9. (A)** The significant gene expression tendencies of profiles 2, 6 and 9. **(B)** The significant enriched GO terms in profiles 2, 6 and 9. The 77 genes in profile 2, which consistently increased in expression, closely correlated with odontogenesis. STC-GO analysis showed genes were involved in cell adhesion, odontogenesis of dentine-containing teeth, establishment or maintenance of cell polarity, cellular component movement, epithelial to mesenchymal transition, regulation of epithelial cell migration, mesenchyme development, BMP signaling pathways, tooth mineralization, positive regulation of apoptosis, enamel mineralization, positive regulation of tooth mineralization, and organ morphogenesis.

To further narrow the target genes with greatly significant differential expression, we placed 2,035 genes in sixteen possible model profiles (Additional file 8) to enrich the expression tendency of the genes using the Series Test of Cluster (STC) analysis. We identified seven patterns of gene expression (profiles 2, 4, 6, 7, 8, 9, and 13, Additional file 9) with significance (*p <* 0.05, Figure 4A). Of these, four patterns were most significant (profiles 4, 7, 8, and 13, *p <* 0.00001, Figure 4A), and the enriched GO terms most significantly associated with these patterns are shown in Figure 4B. The significant tendencies of gene expression can be grouped into three modes: profiles 4, 7, 9, and 13 contained 82, 115, 79, and 284 genes, respectively, whose expression constantly decreased (Figures 4A, and 5A); profile 2 contained 77 genes with constantly increased expression (*p <* 0.05, Figure 5A); profile 6 and 8 contained 144 and 223 genes, respectively, characterized by first decreased then slightly increased expression (*p <* 0.05, Figures 4A, and 5A). The significant enriched GO terms from profiles 2, 6, and 8 are shown in Figure 5B. The lists of assigned genes in each expression profile are included in Additional file 9. Among these patterns, profile 2 had a tendency towards constantly increased gene expression and closely correlated with odontogenesis by STC-GO analysis. The included genes were involved in cell adhesion, odontogenesis of dentine-containing teeth, establishment or maintenance of cell polarity, cellular component movement, epithelial to mesenchymal transition, regulation of epithelial cell migration, mesenchyme development, BMP signaling pathways, tooth mineralization, positive regulation of apoptosis, enamel mineralization, positive regulation of tooth mineralization, organ morphogenesis, etc. STC analysis suggested that down-regulation patterns were predominant and were required for diphyodont morphogenesis, which further verified the array data described above. These gene expression profiles indicated that the assigned genes were involved in dynamic activation and inhibition that may play important roles in diphyodont odontogenesis. Further work is necessary to identify gene regulation patterns.

### Path-net analysis

**Figure 6:**
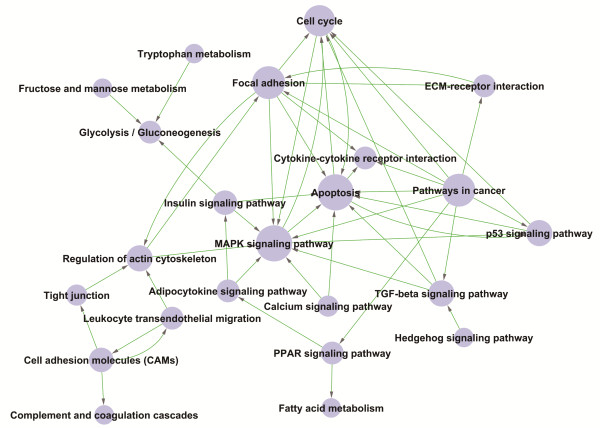
**Significant pathways involving differential genes by path-net construction.** The Path-Net depicted the interactions between the significant pathways of the differentially expressed genes, and was built according to the interactions with pathways in the KEGG database directly and systemically. Cycle nodes represent pathways, the size of nodes represents the power of the interrelation among the pathways, and an arrow between two nodes represents an interaction target between pathways. The more edges of a pathway, the more pathways connecting to it, and the more central role it has within the network.

To further understand the interaction of pathways during diphyodont morphogenesis and screen key pathways with significant role on dental lamina determination, we adopt the strategy to condense information. The pathways were first filtered by STC analysis to enrich 7 significant expression tendencies. Then, an interaction net was built with path-net analysis (Figure 6), according to the graph theory and the interactions among pathways of the KEGG database, to enrich the significant pathways very likely related to diphyodont morphogenesis and identify interactions among the significant pathways involving the up-regulated and down-regulated differentially expressed genes (Fischer’s exact test and the χ^2^ test were used to calculate a P-value to determine significance). As shown in Figure 6, some differentially expressed genes involved in key pathways during diphyodont morphogenesis were identified, including apoptosis, the MAPK signaling pathway, focal adhesion, pathways in cancer, cell cycle, the p53 signaling pathway, regulation of actin cytoskeleton, and the TGF-beta signaling pathway, and so on. Moreover, above-predicted 4 pathways in pathway analysis (TGF-beta signaling pathway, MAPK signaling pathway, Cytokine-cytokine receptor interaction and Hedgehog signaling pathway) and their interaction shown in Figure 6 also played central role. These pathways combined with STC-GO analysis further implied that they likely contributed to dental lamina fate and were required in diphyodont odontogenesis. Thus, the pathway profiles involving differentially expressed genes were identified and provided insights into understanding early diphyodont odontogenesis in miniature pigs. These results, especially pathways related to dental lamina determination, are worthy of further study.

### Signal-net analysis

**Figure 7:**
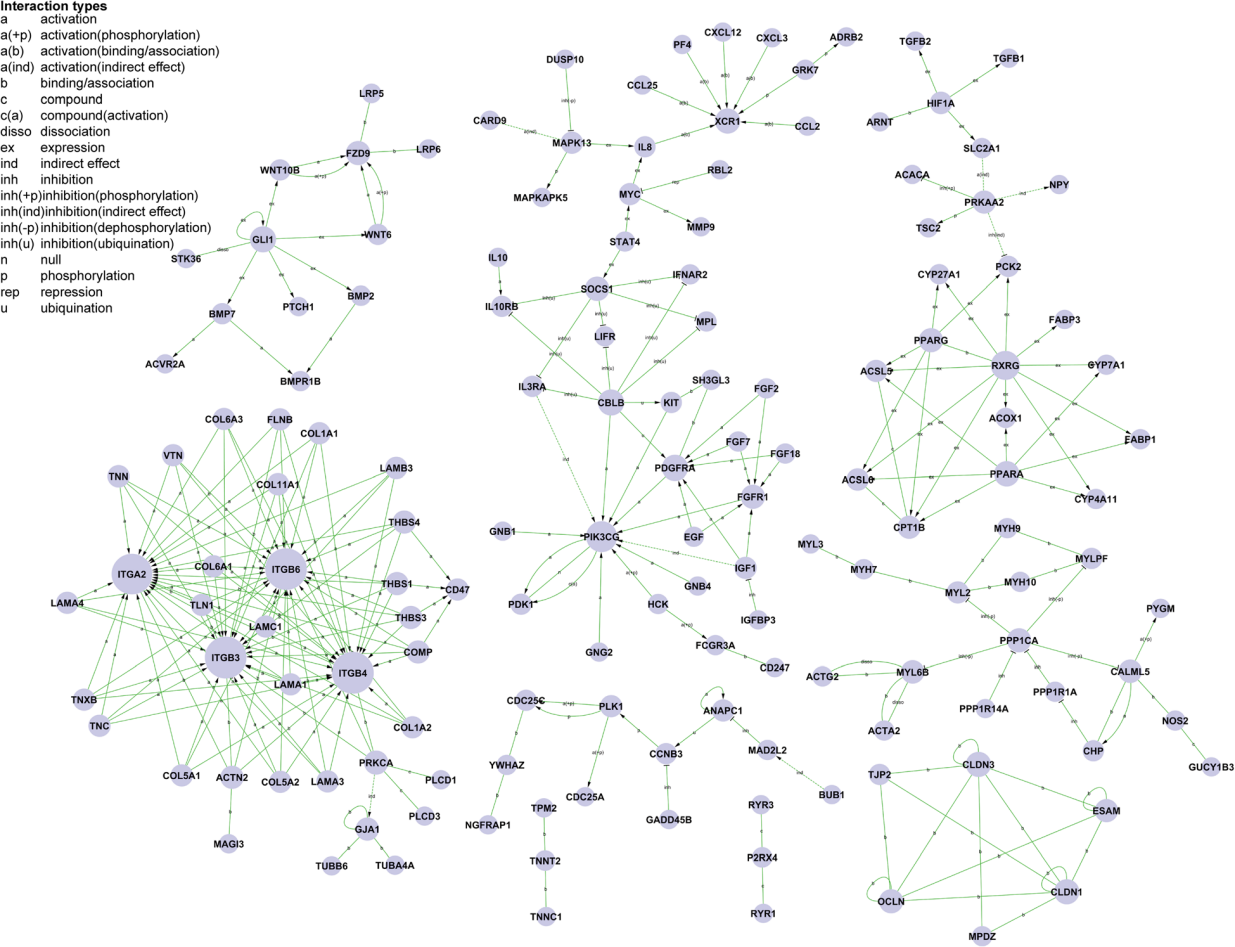
**Gene-gene interaction network.** Genes from significant profiles were analyzed and identified by signal-net construction. Cycle nodes represent genes, the size of nodes represents the power of the interrelation among the genes, and edges between two nodes represent interactions between genes (arrowheads represent targets). The more edges of a gene, the more genes connecting to it, and the more central role it has within the network.

Gene signal transduction networks (Signal-net, based on KEGG database about the interactions between different gene products and the theory of network biology) were established to illustrate the inter-gene signaling between the differentially expressed genes. A total of 151 genes were screened as potential targets for diphyodont morphogenesis (Additional file 10). As shown in Figure 7, the integrin family may play a key role in early diphyodont morphogenesis and odontogenesis, which are involved in ECM-receptor interactions and focal adhesion pathways. Some studies suggested that integrin not only functions to mediate cell adhesion but also serves as an essential element for proliferation during normal homeostasis [44-48]. The results implied niche plays a role in tooth development and provided clues for understanding the molecule mechanisms regulating diphyodont morphogenesis.

## Conclusions

Here, we characterized the temporal gene expression profiles of diphyodont development at early stages in miniature pigs using microarrays. We focused on the significant pathways involved and gene-gene interactions in early stage odontogenesis and identified 151 genes that very likely correlated with diphyodont development. We also identified the GO terms that significantly correlated with the specific genes involved in the molecular function of odontogenesis, including the odontogenesis of dentine-containing teeth, tooth mineralization, regulation of odontogenesis of dentine-containing teeth, and positive regulation of tooth mineralization.

In our study, 2,037 differential genes were screened using a series of bioinformatics analyses of diphyodont morphogenesis. The results indicated that odontogenesis in miniature pigs involves spatio-temporal development of primary teeth and the initiation of secondary dental lamina. There were stage-special gene expression patterns, in which down-regulated genes were predominant on E50 versus E40, while up-regulated genes dominated the morphogenesis of tooth germs on E60 versus E50. In brief, fine-tuning activation and inhibition of gene expression plays an important role in tooth morphology and renewal. These genes form a network and strongly correlate with diphyodont morphogenesis.

Taken together, our results provide a molecule basis for studying pigs, which are excellent animal models for studying teeth development and regeneration. The genes and pathways screened here suggest strong candidates for in-depth studies of the molecular mechanisms of tooth development and diphyodont replacement in miniature pigs. Those results contribute to the understanding of tooth developmental processes in large animal models, which is a fundamental step for investigating the mechanisms of tooth replacement in humans and is applicable to regenerative medicine.

## Methods

### Ethics statement

This study was carried out in strict accordance with the recommendations of the Regulations for the Administration of Affairs Concerning Experimental Animals (Ministry of Science and Technology, China, revised in June 2004). All procedures involving animals described in the present study were reviewed and approved by the Animal Care and Use Committee of Capital Medical University, Beijing, China (Permit Number: CMU-B20100106). All miniature pigs were placed in adjacent identical pens and given continuous access to a standard commercial feed ration and water. All surgery was performed under combination anesthesia, and all efforts were made to minimize suffering. In brief, the timed gestation of the pregnant miniature pigs was determined starting the day following insemination and verified by B-type ultrasonic inspection. The miniature pigs were anesthetized with a combination of 6 mg/kg ketamine chloride and 0.6 mg/kg xylazine, and were sacrificed by over-anesthetization after removing the fetuses by cesarean section.

### Sample preparation

Pregnant Wuzhishan miniature pigs were obtained from the Institute of Animal Science, Chinese Agriculture University, Beijing, China. The staged miniature pig embryos and fetuses were obtained by cesarean section at E40, E50, and E60. The last deciduous molar germs, including the dental lamina in mandibles from the same litter were isolated and pooled, then immerged in 5–10 volumes of RNAlater solution (Ambion, USA) and incubated overnight at 4°C, and then stored at −20°C until needed for total RNA extraction. The morphological stages of the last deciduous molar at E40, E50, and E60 corresponded to the cap, early bell, and late bell stages, respectively, which were verified by serial histological sections.

### Total RNA isolation

Total RNA was extracted from samples at specific stages using TRIZOL Reagent (Life technologies, CA, US) following the manufacturer’s instructions, and then checked for integrity using an Agilent Bioanalyzer 2100 (Agilent technologies, CA US). All solutions used had RNA integrity numbers (RIN) ≥7.0 and 28S/18S ≥0.7. Total RNA was further purified using the RNeasy mini kit and RNase-Free DNase Set (QIAGEN, GmBH, Germany).

### Microarray hybridization and data preprocessing

The GeneChip® Porcine Genome Arrays (Affymetrix, CA, USA), containing 23,937 probe sets to examine 23,256 transcripts (representing 20,201 genes) in a pig, were used for the expression study. The RNA labeling and microarray hybridization were carried out according to the Affymetrix expression analysis technical manual (Biotechnology Corporation, Shanghai, China). Briefly, total RNA was amplified and labeled using the GeneChip 3′IVT express kit (Affymetrix, CA, US), according to the manufacturer’s instructions, to obtain biotin labeled cRNA. Array hybridization and washes were performed as detailed in the manufacturer’s instructions. The arrays were scanned using the GeneChip® scanner 3000 (Affymetrix, CA, US) and Command Console software 3.1 (Affymetrix, CA, US) with default settings. Raw data were normalized using the MAS 5.0 algorithm of Gene Spring software 11.0 (Agilent technologies, CA, US).

### Quantitative real-time RT-PCR

Microarray results were confirmed for a select group of genes (primers used are listed in Additional file 11) by real-time quantitative RT-PCR (qRT-PCR) on the same RNA samples used for the microarray analyses. In brief, total purified RNA was reverse-transcribed using the SuperScriptIII first-Strand synthesis system (Invitrogen). Real time RT-PCR was carried out with SYBR Green PCR mix (Applied Biosystems) and run on the ABI7300 real-time PCR system (Applied Biosystems). Reactions were performed in triplicate in a total volume of 25 μl. Melting curve analysis (60-95°C) was used for assessing amplification specificity. Amplification of swine GAPDH mRNA was used as an endogenous control. Relative expression of each gene was determined using the 2^ΔΔCT^ method. Statistical analyses were performed as described for the microarray data. Pearson’s correlation coefficient was further calculated for each gene using the normalized data to quantify the consistency between microarray experiments and qRT-PCR (*p <* 0.05 and R > 0.9).

### Histochemical and immunohistochemical analysis

Sections were prepared as described previously [34]. Sections were stained with hematoxylin and eosin (H&E) for the tissue morphogenetic study. Immunohistochemistry analyses were performed following the manufacturer’s instructions. Briefly, the sections were deparaffinized and rehydrated, followed by antigen retrieval and incubation with primary antibodies at 4°C overnight. The primary antibodies used were anti-ameloblastin (1:100, Santa Cruz), anti-DSP (1:200, Santa Cruz), and anti-MMP25 (1:200, Santa Cruz). Subsequently, sections were incubated with the corresponding biotinylated secondary antibody and the avidin-biotin complex (ABC kit, Maixin, Fuzhou, China), according to the manufacturer’s protocol. Positive expressions were visualized by DAB (brown). Slides were counterstained with hematoxylin. Images were taken using a microscope (Olympus BX43F) with an attached Olympus DP72 digital camera system.

### Bioinformatics analysis

The details of the bioinformatics analysis (by Genminix Informatics Ltd, Shanghai, China) can be found in Additional file 12. Briefly, to compare the gene expression profiles of all three stages, we first filtered the samples based on the “detection call”. This “call” can either be “present” (p ≤ 0.05), “marginal” (for *p* > 0.05 and ≤0.065) or “absent” (*p* > 0.065). The genes with significant differential expression (a fold change >2) between the 3 different stages of tooth germs were first filtered using significance analysis of microarrays (SAM) software (*p <* 0.05, FDR < 0.05). GO terms (http://www.geneontology.org/) and KEGG pathway (http://www.genome.jp/kegg/) annotations of the differentially expressed genes were found using the DAVID gene annotation tool (http://david.abcc.ncifcrf.gov/). GO and pathway analyses were carried out for significant gene functions and pathway genes. Next, a Series Test of Cluster (STC) analysis was performed for identifying significant expression tendencies and enrichment functions of differentially expressed genes. Network modeling was then performed to determine the interactions between the significant genes (gene signal transduction network), and canonical pathways analysis (Path-Net) determined the interactions among the significant pathways of the differentially expressed genes using Fisher’s exact test. Those analyses were performed on all of the significant genes and on the gene ontology enriched datasets.

### Availability of supporting data

The data sets supporting the results of this article are included within the article and its additional files.

Additional file 1: http://dx.doi.org/10.6070/H4RJ4GC1.

Additional file 2: http://dx.doi.org/10.6070/H4MS3QPR.

Additional file 3: http://dx.doi.org/10.6070/H4H41PC2.

Additional file 4: http://dx.doi.org/10.6070/H4CC0XNB.

Additional file 5: http://dx.doi.org/10.6070/H47M05WJ.

Additional file 6: http://dx.doi.org/10.6070/H43T9F5P.

Additional file 7: http://dx.doi.org/10.6070/H4028PF8.

Additional file 8: http://dx.doi.org/10.6070/H4V9860T.

Additional file 9: http://dx.doi.org/10.6070/H4QJ7F83.

Additional file 10: http://dx.doi.org/10.6070/H4KW5CZP.

Additional file 11: http://dx.doi.org/10.6070/H4G44N7P.

Additional file 12: http://dx.doi.org/10.6070/H4BC3WGH.

## Abbreviations

Dm3: Third deciduous molar; E: Embryonic day; ECM: Extracellular matrix; GO: Gene ontology; qRT-PCR: Real-time reverse transcription-polymerase chain reaction; sdl: Successional dental lamina; STC: Series test of cluster.

## Competing interests

The authors declare that they have no competing interests.

## Authors’ contributions

FW performed the microarray experiments and bioinformatic analyses. SLW designed the research study, analyzed the data, and obtained funding. CW and AL performed qRT-PCR and immunohistochemical analysis. FLW, CMZ, and JJX carried out the animal experiments. ZPF and JQH participated in the design of the study and assisted with the experiments. JX made substantial contributions to the data analysis. FW and SLW drafted the manuscript. All authors read and approved the final manuscript.

## Supplementary Material

Additional file 1 **Detectable transcripts from normalized microarray data.** Table listing all differentially expressed genes with a >2-fold change compared with the other tooth germ stages. Differential genes on E50 versus E40, on E60 versus E50 and E60 versus E40 are shown. Click here for file

Additional file 2 **The up-regulated and down-regulated genes and functions on E50 versus E40.** Table listing the top 20 up-regulated genes (>10-fold differences) and down-regulated genes (>90-fold differences) on E50 versus E40. Click here for file

Additional file 3 **The up-regulated and down-regulated genes and functions on E60 versus E50.** Table listing the top 20 up-regulated genes (>10-fold differences) and top 10 down-regulated genes (>5-fold differences) on E60 versus E50. Click here for file

Additional file 4 **The significant pathways.** Table showing significant pathways and the differential genes involved. Click here for file

Additional file 5 The genes significantly correlated with odontogenesis, from enriched GO categories. Click here for file

Additional file 6 The genes correlated with epithelium determination, derived from enriched GO categories. Click here for file

Additional file 7 **The significant pathways.** Table listing the significant pathways and enriched genes. Click here for file

Additional file 8 **The expression patterns of 2,053 genes analyzed by model profiles.** Figure showing the expression patterns of 2,053 genes were analyzed and summarized by 16 model profiles. Each box represents a model expression profile. The upper number in the profile box is the model profile number and the *p*-value is shown. Seven expression patterns of genes had significant *p*-values (*p* < 0.05), 4 of which had very significant *p*-values (red colored boxes). Click here for file

Additional file 9 **The genes involving significant profiles from STC.** Table listing the genes in each significant profile. The E40, E50, and E60 values represent the time series of gene expression levels for the gene after Log normalized transformation. Click here for file

Additional file 10 **The genes identified by signal-net analysis.** Table listing 151 genes screened as potential targets for diphyodont morphogenesis. Click here for file

Additional file 11 The primer sequences for real-time PCR. Click here for file

Additional file 12 **Supplementary methods.** Including the detailed bioinformatics analysis methods not included in the main text. Click here for file
