# Supplementary figures and images for: Stage-specific differential gene expression profiling and functional network analysis during morphogenesis of diphyodont dentition in miniature pigs, Sus Scrofa
Source: BMC Genomics. 2014 Feb 6;15:103. doi: 10.1186/1471-2164-15-103 (PMC3937075; doi:10.1186/1471-2164-15-103)

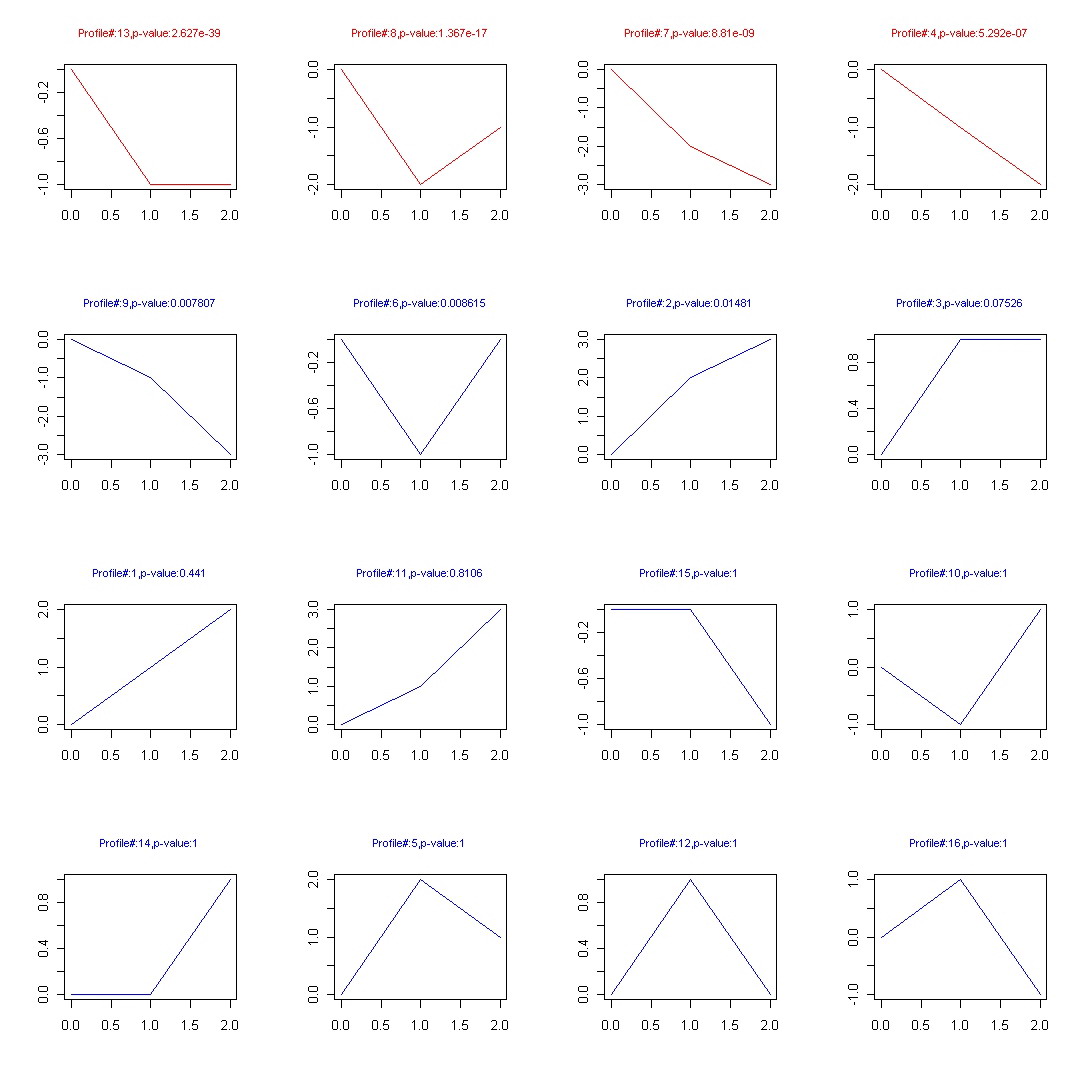

Supplement: Additional file 8 — The expression patterns of 2,053 genes analyzed by model profiles. Figure showing the expression patterns of 2,053 genes were analyzed and summarized by 16 model profiles. Each box represents a model expression profile. The upper number in the profile box is the model profile number and the p-value is shown. Seven expression patterns of genes had significant p-values (p < 0.05), 4 of which had very significant p-values (red colored boxes). [file 1471-2164-15-103-S8.jpeg]
