# Supplementary material for: Stage-specific differential gene expression profiling and functional network analysis during morphogenesis of diphyodont dentition in miniature pigs, Sus Scrofa
Source: BMC Genomics. 2014 Feb 6;15:103. doi: 10.1186/1471-2164-15-103 (PMC3937075; doi:10.1186/1471-2164-15-103)
Supplement: Additional file 12 — Supplementary methods. Including the detailed bioinformatics analysis methods not included in the main text. [file 1471-2164-15-103-S12.doc]

**Additional file 12: Supplementary methods**

**GO analysis**

GO analysis was applied to analyze the main function of the differential expression genes according to the Gene Ontology which is the key functional classification of NCBI [1, 2]. Generally, Fisher’s exact test and $\chi^{2}$test were used to classify the GO category, and the false discovery rate (FDR) [3] was calculated to correct the *P*-value，the smaller the FDR, the small the error in judging the *P*-value. The FDR was defined as$FDR=1-\frac{N_{k}}{T}$, where $N_{k}$ refers to the number of Fisher’s test *P*-values less than$\chi^{2}$test *P*-values. We computed *P*-values for the GOs of all the differential genes. Enrichment provides a measure of the significance of the function: as the enrichment increases, the corresponding function is more specific, which helps us to find those GOs with more concrete function description in the experiment. Within the significant category, the enrichment Re was given by:$\mathrm{Re}=(n_{f}/n)/(N_{f}/N)$where $n_{f}$ is the number of differential genes within the particular category, $n$is the total number of genes within the same category, $N_{f}$is the number of differential genes in the entire microarray, and $N$is the total number of genes in the microarray [4].

**A Series Test of Cluster (STC) analysis**

We selected differential expression genes at a logical sequence according to RVM （Random variance model）corrective ANOVA. In accordance with different signal density change tendency of genes under different situations, we identify a set of unique model expression tendencies. The raw expression values were converted into log_2_ ratio. Using a strategy for clustering short time-series gene expression data, we defined some unique profiles. The expression model profiles are related to the actual or the expected number of genes assigned to each model profile. Significant profiles have higher probability than expected by Fisher’s exact test and multiple comparison tests [5, 6].

**Stc-go analysis**

Go-Analysis is applied to the genes belong to certain specific tendencies. It is used to find the main function of the genes have same expression trend according to the Gene Ontology which is the key functional classification of NCBI. Generally, Fisher’s exact test and $\chi^{2}$test were used to classify the GO category, and the false discovery rate (FDR) was calculated to correct the *P*-value，the smaller the FDR, the small the error in judging the *p*-value. The FDR was defined as$FDR=1-\frac{N_{k}}{T}$, where $N_{k}$ refers to the number of Fisher’s test *P*-values less than$\chi^{2}$test *P*-values. We computed *P*-values for the GOs of all the differential genes. Enrichment provides a measure of the significance of the function: as the enrichment increases, the corresponding function is more specific, which helps us to find those GOs with more concrete function description in the experiment. Within the significant category, the enrichment Re was given by:$\mathrm{Re}=(n_{f}/n)/(N_{f}/N)$ where $n_{f}$ is the number of differential genes within the particular category, $n$is the total number of genes within the same category, $N_{f}$is the number of differential genes in the entire microarray, and $N$is the total number of genes in the microarray.

**Pathway analysis**

Similarly, Pathway analysis was used to find out the significant pathway of the differential genes according to KEGG, Biocarta and Reatome. Still, we turn to the Fisher’s exact test and $\chi^{2}$test to select the significant pathway, and the threshold of significance was defined by *P*-value and FDR. The enrichment Re was calculated like the equation above [7-9].

**Path-net analysis**

The Path-Net was the interaction net of the significant pathways of the differential expression genes, and was built according to the interaction among pathways of the KEGG database to find the interaction among the significant pathways directly and systemically. It could summarize the pathway interaction of differential expression genes under diseases and found out the reason why certain pathway was activated [8].

**Signal-net analysis**

Using java that allows users to build and analyze molecular networks, network maps were constructed. For instance, if there is confirmative evidence that two genes interact with each other, an interaction edge is assigned between the two genes. The considered evidence is the source of the interaction database from KEGG. Networks are stored and presented as graphs, where nodes are mainly genes (protein, compound, etc.) and edges represent relation types between the nodes, e.g. activation or phosphorylation. The graph nature of Networks raised our interest to investigate them with powerful tools implemented in R.

To investigate the global network, we computationally identify the most important nodes. To this **e**nd we turn to the connectivity (also known as degree) defined as the sum of connection strengths with the other network genes:$\text{K}_{i}=\sum_{u\neq i} a_{ui}$. In gene networks, the connectivity measures how correlated a gene is with all other network genes. For a gene in the network, the number of source genes of a gene is called the indegree of the gene and the number of target genes of a gene is its outdegree. The character of genes is described by betweenness centrality measures reflecting the importance of a node in a graph relative to other nodes. For a graph G:(V,E) with n vertices, the relative betweenness centrality$C'_{B}(v)$ is defined by:$C'_{B}(v)=\frac{2}{n^{2}-3n+2}\sum_{\begin{matrix} s\neq v\neq t\in V \\ s\neq t \end{matrix}} \frac{\sigma_{st}(v)}{\sigma_{st}}$ where$\sigma_{st}$ is the number of shortest paths from s to t, and $\sigma_{st}(v)$is the number of shortest paths from s to t that pass through a vertex v [10-14].

**References**

1. **The Gene Ontology (GO) project in 2006**. *Nucleic Acids Res* 2006, **34**(Database issue):D322-326.

2. Ashburner M, Ball CA, Blake JA, Botstein D, Butler H, Cherry JM, Davis AP, Dolinski K, Dwight SS, Eppig JT *et al*: **Gene ontology: tool for the unification of biology. The Gene Ontology Consortium**. *Nat Genet* 2000, **25**(1):25-29.

3. Dupuy D, Bertin N, Hidalgo CA, Venkatesan K, Tu D, Lee D, Rosenberg J, Svrzikapa N, Blanc A, Carnec A *et al*: **Genome-scale analysis of in vivo spatiotemporal promoter activity in Caenorhabditis elegans**. *Nat Biotechnol* 2007, **25**(6):663-668.

4. Schlitt T, Palin K, Rung J, Dietmann S, Lappe M, Ukkonen E, Brazma A: **From gene networks to gene function**. *Genome Res* 2003, **13**(12):2568-2576.

5. Ramoni MF, Sebastiani P, Kohane IS: **Cluster analysis of gene expression dynamics**. *Proc Natl Acad Sci U S A* 2002, **99**(14):9121-9126.

6. Miller LD, Long PM, Wong L, Mukherjee S, McShane LM, Liu ET: **Optimal gene expression analysis by microarrays**. *Cancer Cell* 2002, **2**(5):353-361.

7. Kanehisa M, Goto S, Kawashima S, Okuno Y, Hattori M: **The KEGG resource for deciphering the genome**. *Nucleic Acids Res* 2004, **32**(Database issue):D277-280.

8. Yi M, Horton JD, Cohen JC, Hobbs HH, Stephens RM: **WholePathwayScope: a comprehensive pathway-based analysis tool for high-throughput data**. *BMC Bioinformatics* 2006, **7**:30.

9. Draghici S, Khatri P, Tarca AL, Amin K, Done A, Voichita C, Georgescu C, Romero R: **A systems biology approach for pathway level analysis**. *Genome research* 2007, **17**(10):1537-1545.

10. Jansen R, Greenbaum D, Gerstein M: **Relating whole-genome expression data with protein-protein interactions**. *Genome Res* 2002, **12**(1):37-46.

11. Li C, Li H: **Network-constrained regularization and variable selection for analysis of genomic data**. *Bioinformatics* 2008, **24**(9):1175-1182.

12. Wei Z, Li H: **A Markov random field model for network-based analysis of genomic data**. *Bioinformatics* 2007, **23**(12):1537-1544.

13. Zhang JD, Wiemann S: **KEGGgraph: a graph approach to KEGG PATHWAY in R and bioconductor**. *Bioinformatics* 2009, **25**(11):1470-1471.

14. Spirin V, Mirny LA: **Protein complexes and functional modules in molecular networks**. *Proc Natl Acad Sci U S A* 2003, **100**(21):12123-12128.
